# Supplementary figures and images for: Identification and functional analysis of LecRLK genes in Taxodium ‘Zhongshanshan’
Source: PeerJ. 2019 Aug 13;7:e7498. doi: 10.7717/peerj.7498 (PMC6697044; doi:10.7717/peerj.7498)

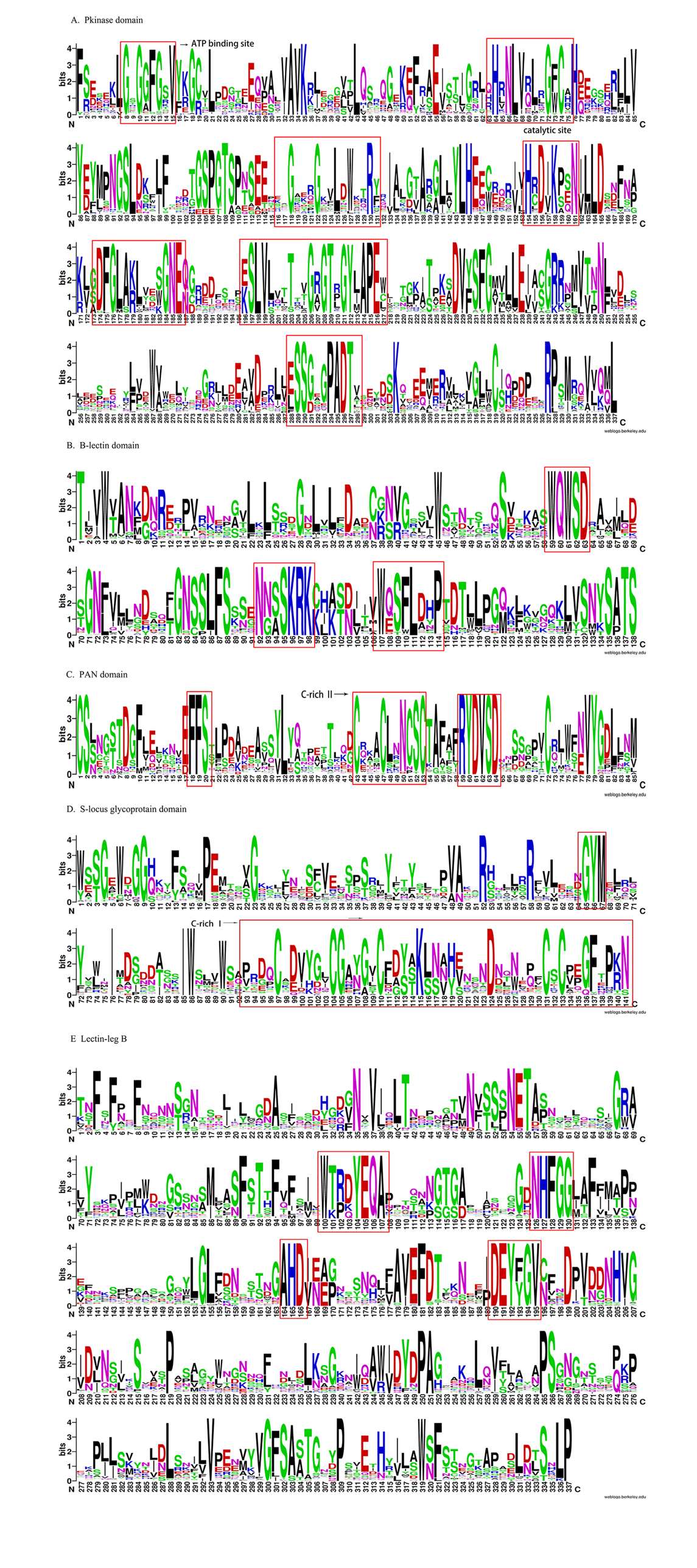

Supplement: File S7 — Conserved motifs were marked using red boxes. Each column in the x-axis is composed of stack of letters where the height of these letters is indicative of the frequency of the letter at that position. The height of the stack is indicative of the sequence conservation. [file peerj-07-7498-s007.tif]
